# Imaging and histological evaluation of the long head of the biceps tendon in the presence of different types of rotator cuff tears

**DOI:** 10.1186/s12891-023-06338-5

**Published:** 2023-03-27

**Authors:** Daisuke Kajiwara, Nobuyasu Ochiai, Eiko Hashimoto, Naoya Hirosawa, Koji Akimoto, Daisuke Nojima, Yohei Shimada, Shohei Ise, Seiji Ohtori

**Affiliations:** 1 Department of Orthopedic Surgery, Chiba University Graduate School of Medicine, 1-8-1 Inohana, Chuo-Ku, Chiba, 260-0856 Japan; 2 Department of Orthopedic Surgery, Chiba Rosai Hospital, Chiba, Japan; 3 Nojima Orthopedics and Internal Medicine Clinic, Tokyo, Japan

**Keywords:** Histologic evaluation, Imaging evaluation, Long head of biceps tendon, Rat model, Rotator cuff tear

## Abstract

**Background:**

A comparison of changes in the long head of the biceps tendon for different types of rotator cuff tears has not been previously performed. Furthermore, the correlation between the thickening and degeneration of the long head of the biceps tendon and the cause of these changes have not been fully clarified. We evaluated the relationship between degenerative changes in the long head of the biceps tendon and rotator cuff tears in a rat model using imaging and histology.

**Methods:**

Ninety-six 12-week-old Sprague–Dawley rats were divided into anterior (subscapularis tear), anterosuperior (subscapularis, supraspinatus, and infraspinatus tears), superior (supraspinatus and infraspinatus tears), and control groups. The long head of the biceps tendon was harvested at 4 or 12 weeks postoperatively. The cross-sectional areas of the intra- and extra-capsular components of the tendon were measured using micro-computed tomography, and the affected/normal ratio of the cross-sectional area was calculated. Masson’s trichrome staining and Alcian blue staining were performed for histologic analysis, with degenerative changes described using the modified Bonar scale. The correlation between the affected/normal ratio and Bonar scores was evaluated.

**Results:**

The affected/normal ratio was higher for the anterior and anterosuperior groups than for the control group at 4 and 12 weeks. The ratio increased for the intra-articular portion in the superior group and for both the intra- and extra-articular portions in the anterior and anterosuperior groups. Degeneration considerably progressed in the anterior and anterosuperior groups compared with the control group from weeks 4 to 12 and was greater in the intra- than in the extra-articular portion. The ratio correlated with extracellular matrix score.

**Conclusions:**

Subscapularis tears were associated with progressive thickening and degeneration of the long head of the biceps tendon at 4 and 12 weeks postoperatively, which was more significant in the intra- than in the extra-articular portion. Histologic evaluation indicated that the extracellular matrix likely caused these degenerative changes.

**Supplementary Information:**

The online version contains supplementary material available at 10.1186/s12891-023-06338-5.

## Background

Rotator cuff tears (RCTs) are a common cause of shoulder pain and dysfunction. Following a RCT, associated damage to the long head of the biceps tendon (LHBT) can be an important source of shoulder pain [1, 2]. Thickening and degenerative changes in the LHBT due to altered mechanical stimulation on the tendon after RCTs have been reported [3, 4]. Moreover, in clinical practice, hour-glass signs of thickening and degeneration of the intra-articular portion of the LHBT are often observed during arthroscopic rotator cuff repair [5]. Tenotomy or tenodesis of the thickened and degenerated LHBT provides good clinical outcomes and pain relief.

Basic research using a rat model has confirmed the thickening and degeneration of the LHBT after RCTs. These changes in the LHBT have been reported for anterosuperior and superior RCTs [6, 7]; however, a comparison of LHBT changes for different types of RCTs has not been previously performed. Furthermore, the correlation between the thickening and degeneration of the LHBT and the cause of these changes have not been fully clarified. Accordingly, this study aimed to evaluate and compare the changes in the LHBT for different types of RCTs in a rat model using imaging and histologic examination. We hypothesized that the thickening and degeneration of the LHBT would depend on the RCT type, being more prominent for RCTs that include a tear of the subscapularis, supraspinatus, and infraspinatus tendons, and that the thickening and degeneration of the LHBT would be correlated.

## Methods

### Statement of ethics

All methods in the present study complied with the relevant guidelines and regulations and are reported in accordance with the ARRIVE guidelines (https://arriveguidelines.org) for the reporting of animal experiments. In particular, this study was conducted in accordance with the Guideline for Animal Experimentation of Chiba University; Act on Welfare and Management of Animals; Fundamental Guidelines for Proper Conduct of Education, Culture, Sports, Science and Technology by Chiba University; and Institutional Animal Care and Use Committee of Animal Experimentation at Chiba University. Furthermore, this study was approved by our Institutional Review Board (approval number: 2–84) and adhered to the National Institutes of Health’s and our university’s Guidelines for the Care and Use of Laboratory Animals (1996 Revision).

### Animal model of RCT

**Fig. 1:**
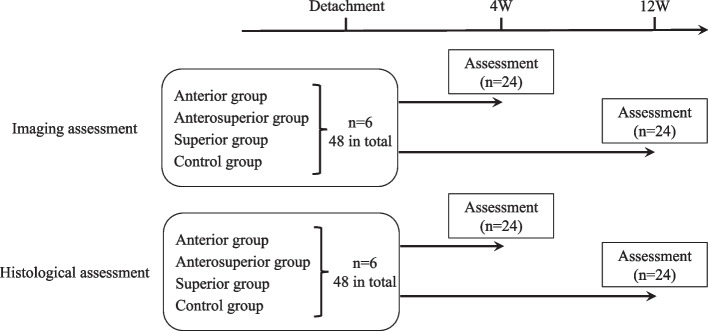
Group classification

Ninety-six adult (12-week-old) Sprague–Dawley rats (Japan SLC Inc., Hamamatsu, Shizuoka, 380–460 g) were used in this study. The rats were divided into two groups according to the method of evaluation, which included imaging or histologic examination. The following three RCT types were created on the left side, with an equal number of animals in each group: anterior group, subscapularis tear only; anterosuperior group, subscapularis, supraspinatus, and infraspinatus tear; superior group, supraspinatus and infraspinatus tear; and control group, no tear. Group classifications are shown in Fig. 1.

### Surgical procedure

**Fig. 2:**
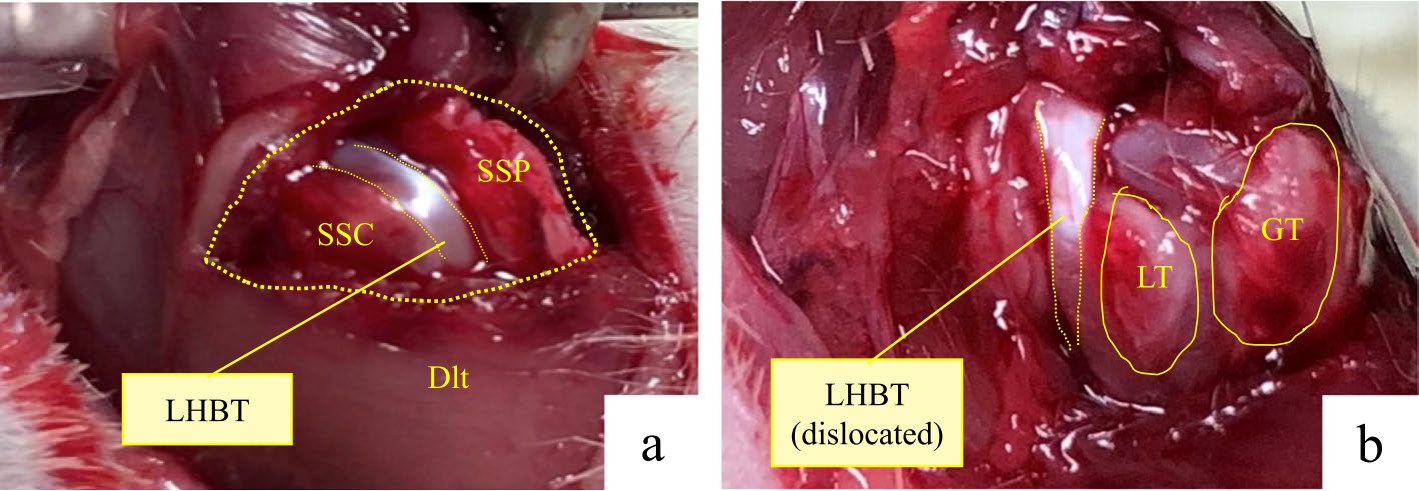
Rotator cuff tear model (left shoulder). **a** LHBT exposure. **b** Case from the anterosuperior group with LHBT dislocation. *LHBT* long head of the biceps tendon, *Dlt* deltoid, *SSP* supraspinatus, *SSC* subscapularis, *GT* greater tuberosity, *LT* lesser tuberosity, *AS* anterosuperior

Procedures were performed with animals in the prone position, and intraperitoneal anesthesia was used (medetomidine [Domitor, Orion Corp., Finland] at 0.15 mg/kg, midazolam [Dormicum, Astellas Pharma Inc., Japan] at 2 mg/kg, and butorphanol [Vetorphale, Meiji Seika Pharma Co., Ltd. Japan] at 2.5 mg/kg) [8]. With the left foreleg in external rotation, a 2-cm skin incision was made, in the proximal to the distal direction, over the midlateral corner of the acromion. The deltoid was sharply dissected in an anterior to distal direction 5 mm distal to the acromion and in a proximal to distal direction 5 mm proximal to the point 15 mm distal from the edge of the acromion. The T-shaped cut deltoid muscle was divided, and the rotator cuff was identified. The rotator cuff was visualized at its insertion into the humerus (Fig. 2a). The tendons were completely dissected from their point of attachment on the greater or lesser tuberosity, and a 5-mm enthesis was resected for each tendon to prevent tendon healing.

The tendon sheath was cut longitudinally to expose the LHBT (Fig. 2b). The subscapularis tendon was detached from its point of attachment on the lesser tuberosity in the anterior group. In the superior group, the supraspinatus and infraspinatus tendons were detached at their point of attachment on the greater tuberosity. In the anterosuperior group, the subscapularis, supraspinatus, and infraspinatus tendons were detached at their point of attachment on the lesser and greater tuberosities. The LHBT was easily dislocated by the internal and external rotation of the shoulder in the anterior and anterosuperior groups. In the control group, only the rotator cuff tendon was exposed. The deltoid muscle and the skin were sutured using 4–0 nylon sutures in all groups (Nescosuture; Alfresa Pharma Corp., Japan). The rats were allowed unrestricted cage activity [9, 10]. All the rats were observed for signs of infection or other complications throughout the study period.

### Imaging assessment

**Fig. 3:**
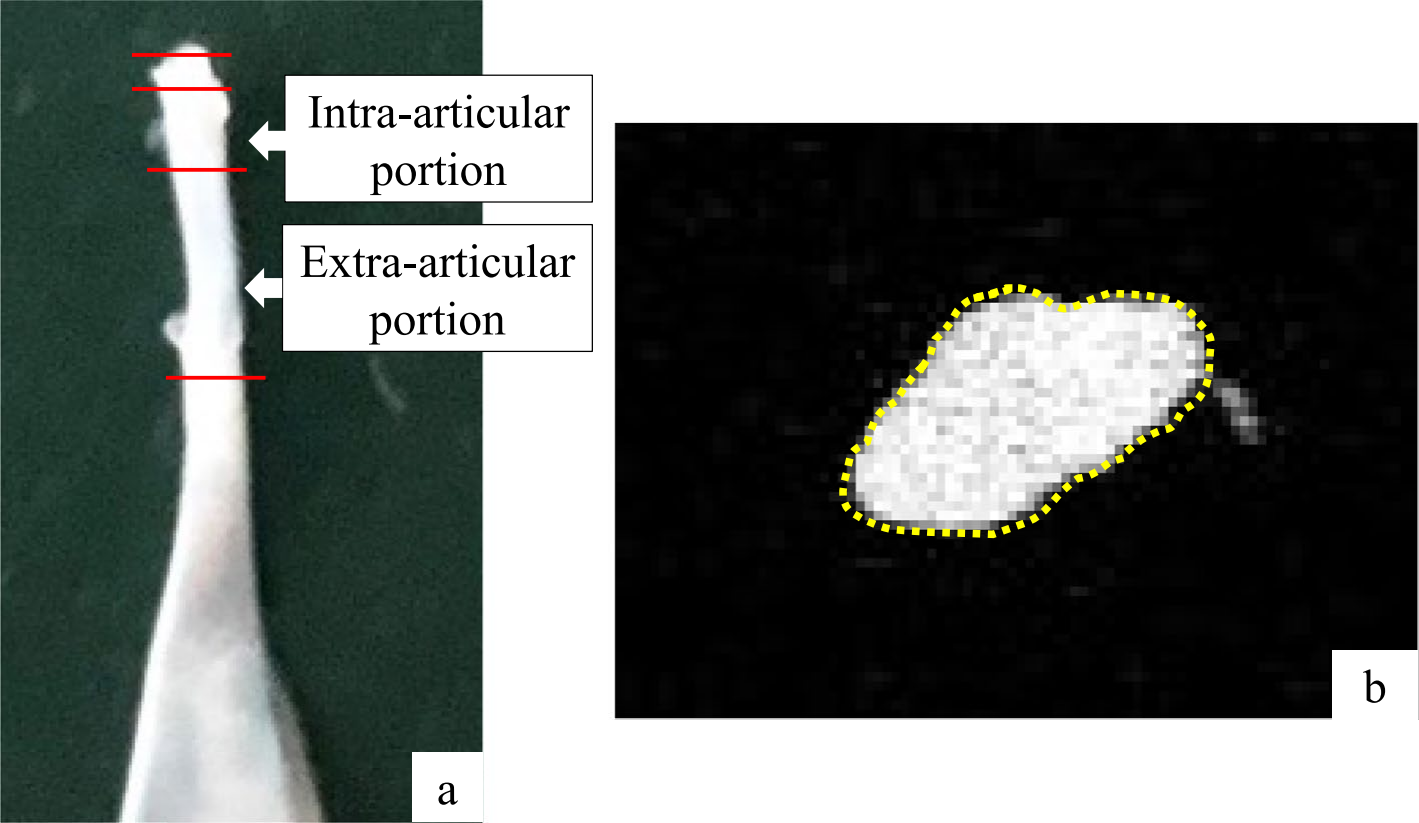
LHBT imaging (left shoulder). **a** Harvested LHBT. **b** Cross-sectional micro-computed tomography image of the LHBT. *LHBT* long head of the biceps tendon

In each group, the rats were euthanized at 4 (*n* = 6) and 12 (*n* = 6) weeks postoperatively by transcardial perfusion of 4% paraformaldehyde in phosphate buffer. The LHBT, including the muscle, was removed bilaterally and stored in 10% neutral buffered formalin solution. The cross-sectional area (CSA) of the LHBT was measured using high-resolution micro-computed tomography (Latheta LCT-100 scanner; Hitachi-Aloka Medical Ltd., Tokyo, Japan) (Fig. 3a). The intra-articular portion of the tendon was defined as 1.5–3.5 mm from the point of attachment of the LHBT on the glenoid and the extra-articular portion as 3.5–8.5 mm from point of attachment, as previously described [6] (Fig. 3b). A 30-G needle (Terumo, Japan) was used as a marker of the 1.5-, 3.5-, and 8.5-mm positions on the tendon. According to a previous study that measured the CSA of the entire LHB at nine different locations [3], the CSA of the intra-and extra-articular portions of the LHBT in all groups was measured at five points at an interval of 0.4 mm for the intra-articular portion and 1.0 mm intervals for the extra-articular portion respectively. The average CSA of the affected/normal (A/N) side ratio was calculated.

### Histologic assessment

For histologic analysis, six rats from each group were euthanized at 4 and 12 weeks postoperatively. The left LHBT was harvested, fixed in 10% neutral buffered formalin solution, and embedded in paraffin. Five-µm LHBT sections were cut parallel to the LHBT fibers, stained with Masson’s trichrome and Alcian blue (Supplementary Methods), and evaluated under digital microscopy (Axioskop 2 Plus, Carl Zeiss, Germany; 200 × magnification). Degenerative changes in the LHBT were evaluated semi-quantitatively using the modified Bonar scale [9–11] in a blinded and randomized manner. This scoring system consists of four subcategories: tenocytes, ground substance, collagen, and vascularity. Each category is scored on a 3-point scale (0–3), with a maximum total score of 12 points and with higher scores indicating greater degenerative changes (Table 1). Tenocytes, collagen, and vascularity were evaluated by Masson’s trichrome staining, with the ground substance evaluated by Alcian blue staining. Degeneration of the extracellular ground substance is shown as mucoid-like substrates, glycosaminoglycans, and proteoglycans; the score across all subcategories was summed. Ten slices were obtained from random locations in the intra- and extra-articular portions of the tendon for evaluation as previously described [9, 10], with a total of 50 sections included in the analysis for each animal.

**Table 1:** Semiquantitative scoring system using the modified Bonar scale [11]

Subcategory	Grade			
	**0**	**1**	**2**	**3**
Tenocytes	Inconspicuous elongated spindle shaped	Increased roundness: Nucleus becomes more ovoid to round in shape	Increased roundness and size: The nucleus is round	Nucleus is round, large
Ground substance	No stainable ground substance	Stainable mucin between fibers but bundles still discrete	Stainable mucin between fibers with loss of clear demarcation of bundles	Abundant mucin throughout with inconspicuous collagen staining
Collagen	Collagen arranged in tightly cohesive well demarcated bundles with a smooth dense bright homogeneous polarization pattern with normal crimping	Diminished fiber polarization: Separation of individual fibers with maintenance of demarcated bundle	Bundle changes: Separation of fibers with loss of demarcation of bundles giving rise to expansion of the tissue overall and clear loss of normal polarization pattern	Marked separation of fibers with complete loss of architecture
Vascularity	Inconspicuous blood vessels coursing between bundles	Occasional cluster of capillaries, less than 1 per 10 high power fields	1–2 clusters of capillaries per 10 high power fields	Greater than 2 clusters per 10 high power fields

### Statistical analysis

All statistical analyses were performed using JMP Pro 15 software (SAS Institute Inc., USA). The results are presented as mean ± standard deviation for each group. Differences between groups were compared using Tukey’s honestly significant difference test, and changes over time and intra-/extra-articular differences were compared using Student’s t-test. The correlation between the thickening and degeneration of the LHBT was evaluated by calculating Spearman’s rank correlation between the A/N ratio of the CSA and subcategories and the total modified Bonar scale scores. Significance was defined as a *p*-value of < 0.05.

## Results

### Imaging results

No complications occurred during or after surgery including infection or epiphyseal fractures. The A/N ratio of the CSA was greater for the superior, anterior, and anterosuperior groups than for the control group at 4 and 12 weeks postoperatively in both the intra- and extra-articular portions of the tendon. Compared to the control group, the increase in the A/N ratio was significant in the anterosuperior group at 4 weeks (intra-articular portion, *p *= 0.0251; extra-articular portion, *p* = 0.0212) and in the anterior and anterosuperior groups at 12 weeks (anterior group, intra-articular portion, *p* = 0.0291, and extra-articular portion, *p *= 0.0417; anterosuperior group, intra-articular portion, *p* = 0.0191, and extra-articular portion, *p *= 0.0211). The A/N ratio increased from week 4 to 12 in the superior, anterior, and anterosuperior groups for both the intra-articular (superior, *p* = 0.0429; anterior, *p* = 0.0421; and anterosuperior, *p* = 0.0327) and extra-articular (anterior, *p* = 0.0328; anterosuperior, *p* = 0.0291) portions of the tendon. Differences were found between the intra- and extra-articular portions in all groups (Tables 2 and 3).

**Table 2:** A/N ratio of the CSA of the LHBT at 4 weeks postoperatively

	**A/N ratio of CSA**	
	**Itnra-articular portion**	**Extra-articular portion**
Control group (no tear)	1.1 ± 0.2	1.1 ± 0.1
Superior group (SSP + ISP tear)	1.5 ± 0.3	1.4 ± 0.4
Anterior group (SSC tear)	1.7 ± 0.5	1.5 ± 0.5
Anterosuperior group (SSC + SSP + ISP tear)	2.3 ± 0.5^a^	2.0 ± 0.3^a^

*A/N* Affected/normal, *CSA* Cross-sectional area, *SSP* Supraspinatus, *ISP* Infraspinatus, *SSC* Subscapularis, *LHBT* Long head of the biceps tendon

^a^Significant difference from the control group

**Table 3:** A/N ratio of the CSA of the LHBT at 12 weeks postoperatively

	**A/N ratio of CSA**	
	**Intra-articular portion**	**Extra-articular portion**
Control group (no tear)	1.0 ± 0.3	1.1 ± 0.3
Superior group (SSP + ISP tear)	2.0 ± 0.5^c^	1.5 ± 0.4
Anterior group (SSC tear)	2.5 ± 0.5^c^	2.4 ± 0.6^a,c^
Anterosuperior group (SSC + SSP + ISP tear)	2.9 ± 0.5^a,c^	2.6 ± 0.5^a,b,c^

*A/N* Affected/normal, *CSA* Cross-sectional area, *SSP* Supraspinatus, *ISP* Infraspinatus, *SSC* Subscapularis, *LHBT* Long head of the biceps tendon

^a^Significant difference from the control group

^b^Significant difference from the superior group

^c^Significant difference from 4 weeks postoperatively

### Histologic results

#### Modified bonar scale

**Fig. 4:**
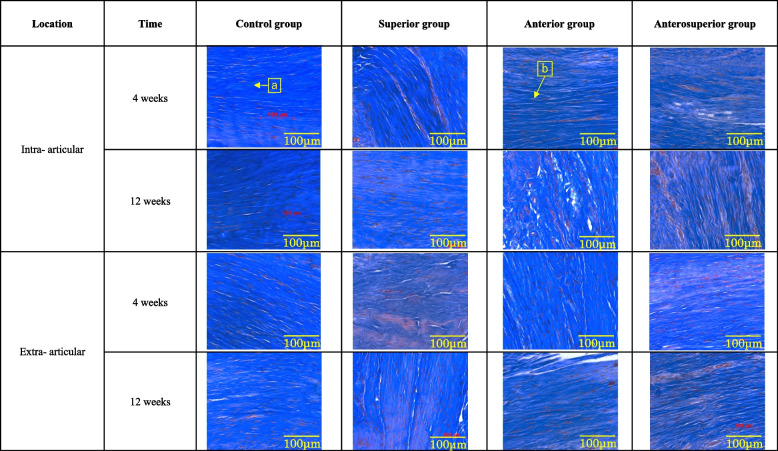
Histologic assessment of LHBT degeneration by Masson’s trichrome staining. The modified Bonar scale was used for the analysis: (**a**) tenocytes, (**b**) collagen, and (**c**) vascularity. *LHBT* long head of the biceps tendon

**Fig. 5:**
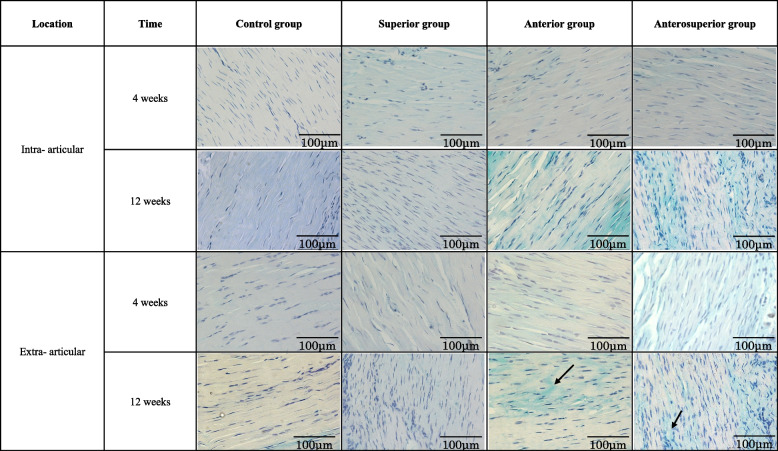
Histologic assessment of the LHBT by Alcian blue staining. The modified Bonar scale was used for the analysis, with the arrow indicating the ground substance. *LHBT* long head of the biceps tendon

There were no degenerative changes in the intra- and extra-articular portions of the tendon at either time point in the control group. Degenerative changes were observed in the three RCT groups, in both the intra- and extra-articular portions of the tendon, at 4 weeks postoperatively, including diminished fiber polarization and rounding of the cell nucleus. Rounder and larger nuclei, clusters of capillaries, and marked separation of fibers, with complete loss of the tendon architecture, appeared in the RCT groups, particularly noticeable in the intra-articular portion of the tendon in the anterosuperior group at 12 weeks postoperatively (Figs. 4 and 5). The total modified Bonar scale scores are shown in Tables 4 and 5. The between-group comparison revealed a greater degeneration in the superior group (intra-articular portion, *p* = 0.0177 at 4 weeks and *p* = 1.44 e^−5^ at 12 weeks; extra-articular portion, *p* = 0.000487 at 12 weeks), anterior group (intra-articular portion, *p* = 1.96 e^−5^ at 4 weeks and *p* = 1.29 e^−5^ at 12 weeks; extra-articular portion, *p* = 4.57 e^−5^ at 12 weeks), and anterosuperior group (intra-articular portion, *p* = 8.77 e^−6^ at 4 weeks and *p* = 2.52 e^−8^ at 12 weeks; extra-articular portion, *p* = 0.0008 at 4 weeks and *p* = 9.04 e^−6^ at 12 weeks) than in the control group. There were no significant differences between the anterior and anterosuperior groups at 4 and 12 weeks, or between the intra- and extra-articular portions (intra-articular portion, *p* = 0.232 at 4 weeks and *p* = 0.130 at 12 weeks; extra-articular portion, *p* = 0.0894 at 4 weeks and *p* = 0.149 at 12 weeks). The scores progressed from weeks 4 to 12 in the anterior and anterosuperior groups (anterior group, intra-articular portion, *p* = 0.0238, and extra-articular portion, *p* = 0.0197; anterosuperior group, intra-articular portion, *p* = 8.63 e^−5^, and extra-articular portion, *p* = 0.0233). The extent of degeneration was greater in the intra- than in the extra-articular portions of the tendon in the anterior and anterosuperior groups at 12 weeks (*p* = 0.0312 and 0.000465, respectively).

**Table 4:** Total modified Bonar scale score for the LHBT at 4 weeks postoperatively

	**Modified Bonar scale score**	
	**Intra-articular portion**	**Extra-articular portion**
Control group (no tear)	1.1 ± 0.6	1.1 ± 0.6
Superior group (SSP + ISP tear)	2.1 ± 0.8^a^	2.3 ± 1.0
Anterior group (SSC tear)	3.3 ± 0.7^a^	2.1 ± 0.8
Anterosuperior group (SSC + SSP + ISP tear)	3.8 ± 0.9^a,b^	3.0 ± 1.1^a^

*A/N* Affected/normal, *CSA* Cross-sectional area, *SSP* Supraspinatus, *ISP* Infraspinatus, *SSC* Subscapularis, *LHBT* Long head of the biceps tendon

^a^Significant difference from the control group

^b^Significant difference from the superior group

**Table 5:** Total modified Bonar scale score for the LHBT at 12 weeks postoperatively

	**Modified Bonar scale score**	
	**Intra-articular portion**	**Extra-articular portion**
Control group (no tear)	1.1 ± 0.6	1.3 ± 0.9
Superior group (SSP + ISP tear)	3.2 ± 0.7^a^	3.3 ± 0.9^a^
Anterior group (SSC tear)	5.6 ± 1.1^a,c,d^	3.6 ± 0.7^a,c^
Anterosuperior group (SSC + SSP + ISP tear)	6.8 ± 1.3^a,b,c,d^	4.3 ± 0.9^a,c^

*A/N* Affected/normal, *CSA* Cross-sectional area, *SSP* Supraspinatus, *ISP* Infraspinatus, *SSC* Subscapularis, *LHBT* Long head of the biceps tendon

^a^Significant difference from the control group

^b^Significant difference from the superior group

^c^Significant difference from 4 weeks

^d^Significant from the extra-articular portion

**Fig. 6:**
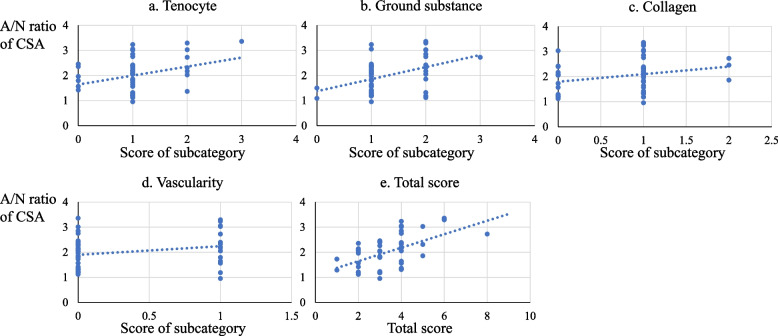
Scatter plots of A/N ratio of CSA of the LHBT and modified Bonar scale score. **a** Tenocyte, (**b**) ground substance, (**c**) collagen, (**d**) vascularity, (**e**) total score. *A/N* affected/normal, *CSA* cross-sectional area, *LHBT* long head of the biceps tendon

A significant correlation was found between the A/N ratio of the CSA and each subgroup Bonar scale score for the extracellular matrix (*r* = 0.41989; *p* = 0.00360) and total scale score (*r* = 0.54403; *p* = 8.77 e^−5^) (Table 6 and Fig. 6).

**Table 6:** Correlation coefficients between the A/N ratio of the LHBT CSA and modified Bonar scale score

Modified Bonar score	Subcategories				Total score
	**Tenocyte**	**Ground substance**	**Collagen**	**Vascularity**	
Correlation coefficients	0.3295	0.41989	0.26227	0.25437	0.54403
*P* value	0.02512968	0.00359691	0.07797324	0.08771918	8.7727e^−5^

*A/N* Affected/normal, *CSA* Cross-sectional area, *LHBT* Long head of the biceps tendon

## Discussion

In this study, imaging and histologic evaluation of the LHBT in a rat model of RCT revealed thickening and degeneration of both the intra- and extra-articular portions of the tendon. Furthermore, our results also showed significant changes in the intra-articular portion of the anterosuperior group. Clinically, patients with an anterosuperior RCT have a greater CSA of the LHBT, measured by ultrasonography in the bicipital groove (extra-articular portion), than patients with a medium-sized posterosuperior RCT [12]. Separate assessments of the intra- and extra-articular portions of the LHBT for subscapularis tears have been performed clinically; nonetheless, anterosuperior-type RCTs, including subscapularis, supraspinatus, and infraspinatus, and isolated subscapularis tears, have not been previously assessed [6]. Peltz et al. created a rat model of supraspinatus tears, supraspinatus and subscapularis tears, and supraspinatus and infraspinatus tears, measuring the CSA of the LHBT at 4 and 8 weeks postoperatively, but did not consider the intra- the extra-articular portions separately for between-group comparisons [6].

To our knowledge, our study is the first to compare the CSA of the LHBT among RCT types, showing a greater CSA (tendon thickening) in the anterior and anterosuperior groups than in the other groups. Our findings of a remarkable thickening of the LHBT with an anterosuperior RCT are consistent with previously reported clinical data [12]. Takahashi and Arai reported dislocation and increased instability of the LHBT with subscapularis tears, particularly with anterior tears of the subscapularis and supraspinatus tendons [12, 13], which lead to thickening of the tendon [3]. In their report of 71 RCTs with dislocation or subluxation of the LHBT, Walch et al. reported that 69 (97.2%) cases had a complete or partial tear of the subscapularis tendon, underlining the risk of LHBT instability with this type of RCT [14]. In our study, LHBT instability increased in subscapularis tears, with a greater CSA and progressive thickening over time. LHBT thickening associated with an RCT has previously been shown in both the intra- and extra-articular portions of the tendon using cadaveric studies, arthroscopic evaluation, or ultrasound examination [3, 12]. In their cadaveric study, Aizawa et al. reported significant LHBT thickening of both the intra- and extra-articular portions of the tendon in the bicipital groove [3]. Takahashi et al. evaluated the CSA of the extra-articular portion of the LHBT, by ultrasonography, before arthroscopic RCT repair, showing a thickening of the tendon in the extra-articular portion before surgery [12]. In our study, by comparing RCT-related changes in both the intra- and extra-articular portions of the tendon, we did not identify a difference in tendon thickening between the two portions. As the assessment of the intra-articular portion of the LHBT is difficult using preoperative (magnetic resonance and ultrasound) imaging, our findings indicate that assessment of the extra-articular portion of the tendon may predict changes in the intra-articular portion. The subscapularis tendon provides inferior support to the LHBT, fixing the tendon within the bicipital group and, thus, resisting an anteromedial dislocation of the tendon [13]. As such, subscapularis tears considerably affect the stability of the LHBT [12]. LHBT instability has been observed during arthroscopic RCT repair, particularly with subscapularis tears [15]. This finding substantiates our findings of a greater A/N ratio of the tendon CSA in the anterior and anterosuperior groups than in the superior and control groups. Our study further underlined the progressive degeneration of the LHBT from 4 to 12 weeks postoperatively in the anterior and anterosuperior groups.

The histologic features of tendinopathy have previously been evaluated using the modified Bonar scale for the Achilles tendon, patellar tendon, rotator cuff, and LHBT [9, 10, 16, 17], with findings consistent with those of our study, including the changes in tenocyte morphology and an increase in the extracellular matrix [11, 18, 19]. The incidence of degenerative LHBT lesions correlates with the size and severity of RCT [6], with greater degeneration, including greater disorganization of collagen fibers and higher proteoglycan content, for the intra-articular portions rather than for the extra-articular portions of the LHBT [20]. Leffert et al. proposed that hypertrophy represents a mechanism of functional compensation for RCTs [21] to improve the capacity of the LHBT to compress the humeral head in the glenoid cavity during shoulder movements [22]. Boileau et al. reported that an hour-glass degenerative lesion of the LHBT resulted from multiple factors, including functional hypertrophy due to a deficient superior rotator cuff, the inflammatory process resulting from anterosuperior impingement of the tendon under the coracoacromial arch, and an inflammatory process due to repetitive friction of the tendon with the narrow bicipital groove [5]. Takahashi et al. also explained that shoulder instability caused by RCTs increases the mechanical stress on the LHBT, causing hypertrophy [12]. Intra-articular mechanical stimuli, such as compression, shearing, and friction, are more common than extra-articular stimuli and are thought to be the cause of intra-articular degeneration in clinical situations [4].

To our knowledge, our study is the first to compare degeneration between the intra- and extra-articular portions of the LHBT. In our rat model, significant degeneration of the intra-articular portion of the LHBT was observed in the anterior and anterosuperior groups, but not in the superior and control groups. The causes of hypertrophic LHBTs and their association with LHBT degeneration have not been fully clarified. Our study revealed a correlation between the A/N ratio of the CSA of the LHBT and the Bonar score (degeneration), with the strongest correlation between the A/N ratio and the Bonar subgroup score for the extracellular matrix and the total score. This is consistent with the findings of Joseph et al. who reported a significant increase in the extracellular matrix of thickened LHBTs [23]. Therefore, although our results could not possibly clarify the cause of the increase in extracellular matrix, the increase may have some relationship to the increase in CSA of the LHBT, and it is possible that the increase in extracellular matrix may be one of the causes of LHBT thickening. Further studies are necessary to clarify the molecular mechanisms underlying the observed changes in the extracellular matrix of the LHBT.

The limitations of our study need to be acknowledged. First, RCTs were surgically created in our model, whereas RCTs in clinical practice are generally caused by tendon degeneration. Second, the anatomy and function of the rat shoulder differ from those of the human shoulder, although previous studies have identified this model as being appropriate to investigate rotator cuff and LHBT pathology [7, 24, 25]. Lastly, in addition to molecular studies, immunohistochemistry, and RNA microarray analysis may provide additional information on the underlying mechanisms.

## Conclusions

The thickening and degeneration of the LHBT were observed after RCTs; and were greatest in the subscapularis and supraspinatus, and infraspinatus tendon tears. Furthermore, the observed changes included those with only a subscapularis tendon tear. Thickening and degenerative changes considerably progressed from 4 to 12 weeks postoperatively, and were more noticeable for the intra- than for the extra-articular portion of the LHBT. Histologic examination revealed that these changes most likely resulted from changes in the extracellular matrix of the tendon.

## Supplementary Information

**Additional file 1:** **Supplementary Methods.**

## Data Availability

The data used and/or analyzed during the current study are available from the corresponding author (Daisuke Kajiwara, daisuke.0108.kajiwara@gmail.com) on reasonable request.

## Abbreviations

A/N: Affected/normal
CSA: Cross-sectional area
LHBT: Long head of the biceps tendon
RCTs: Rotator cuff tears
