# Supplementary material for: Imaging and histological evaluation of the long head of the biceps tendon in the presence of different types of rotator cuff tears
Source: BMC Musculoskelet Disord. 2023 Mar 27;24:230. doi: 10.1186/s12891-023-06338-5 (PMC10045614; doi:10.1186/s12891-023-06338-5)
Supplement: Supplementary file 1 — Additional file 1: Supplementary Methods. [file 12891_2023_6338_MOESM1_ESM.docx]

**Supplementary Methods**

***Dyeing method***

For Masson’s trichrome staining, sections were deparaffinized and saturated with a dye-mordant (10% potassium dichromate + 10% trichloroacetic acid) for 30 min. Subsequently, the sections were stained with Carazzi's hematoxylin for 45 min, 0.75% Orange G solution for 2 min, Masson stain solution B for 20 min, 2.5% phosphotungstic acid solution for 10 min, and aniline blue solution for 20 min. For Alcian blue staining, the sections were deparaffinized and saturated with 3% acetic acid for 1 min. Subsequently, the sections were stained with Alcian blue solution (pH, 2.5) for 20 min, saturated with 3% acetic acid for 3 min, and stained with Mayer’s hematoxylin solution for 2 min. Finally, all sections were dehydrated and covered with a glycerol coverslip.
